# Appendiceal goblet cell adenocarcinoma synchronous with ascending colon adenocarcinoma and severe anemia: A case report

**DOI:** 10.1097/MD.0000000000043452

**Published:** 2025-07-18

**Authors:** Yi-Hu Mao, Shu-Jun Li, Qi Jia, Qian Zeng, Jian Yang, Cai-Jun Yang, Qi Pu, Ting Zhang, Xue-Ping Liu, Li Jiang

**Affiliations:** a Department of Gastrointestinal Surgery, The People’s Hospital of Lezhi, Lezhi, China; b Department of Pathology, The People’s Hospital of Lezhi, Lezhi, China.

**Keywords:** anemia, appendiceal goblet cell adenocarcinoma, colorectal cancer, right hemicolectomy, synchronous

## Abstract

**Rationale:**

Appendiceal goblet cell adenocarcinoma (AGCA) synchronous with colorectal cancer (CRC) is extremely rare, with only a few cases reported in the literature. The oncogenic mechanisms, diagnosis, and treatment of the coexistence of AGCA and CRC face significant challenges. This case report describes a patient diagnosed with ascending colon adenocarcinoma and severe anemia. Pathological examination after radical right hemicolectomy revealed goblet cell adenocarcinoma in the appendix. The patient subsequently received postoperative chemotherapy, and the prognosis was favorable.

**Patient concerns:**

A 61-year-old female presented with abdominal pain and distension lasting over 6 months and was admitted to the hospital with a red blood cell count of 2.5 × 10^9^/L and hemoglobin levels of 55.00 g/L.

**Diagnoses:**

Abdominal computed tomography demonstrated thickening of the ascending colon, raising suspicion of a colon tumor, and no abnormality in the appendix. Colonoscopy confirmed the presence of a mass in the ascending colon, and the biopsy results suggested adenocarcinoma. The preoperative diagnosis was ascending colon adenocarcinoma with severe anemia.

**Interventions:**

After a multidisciplinary discussion, the patient underwent radical right hemicolectomy under general anesthesia.

**Outcomes:**

Postoperative pathology revealed ascending colon adenocarcinoma and AGCA. The patient subsequently received postoperative chemotherapy, and the prognosis was favorable.

**Lessons:**

Synchronous AGCA with CRC are rare. The oncogenic mechanisms, diagnosis, and treatment of the coexistence of AGCA and CRC face significant challenges, and radical tumor resection combined with postoperative chemotherapy is an important treatment strategy. We contend that for patients with AGCA combined with ascending colon adenocarcinoma, radical right hemicolectomy is a reasonable approach, as it allows for the simultaneous radical resection of tumors in both locations.

## 1. Introduction

Appendiceal goblet cell adenocarcinoma (AGCA) is a extremely rare malignancy.^[1]^ Its incidence is approximately 1–5/1,00,00,000, and AGCA accounts for approximately 15% of all appendiceal tumors.^[2]^ AGCA is difficult to diagnose preoperatively and is usually diagnosed incidentally after appendectomy. Although colorectal adenocarcinoma is more common, coexistence of AGCA and colorectal cancer (CRC) is extremely rare.^[3]^ We report a case of AGCA synchronous with ascending colon adenocarcinoma and severe anemia. No lesions were found in the appendix during the preoperative examination; however, postoperative pathological examination revealed goblet cell adenocarcinoma of the appendix.

## 2. Case report

### 2.1. Patient information

A 61-year-old female, presented with abdominal pain and distension lasting over 6 months and was admitted to the hospital on February 19, 2021. The patient had no history of any disease or surgery.

### 2.2. Physical examination

The patient’s mental state was poor and his skin appeared pale; however, there were no signs of jaundice or cardiopulmonary abnormalities. The vital signs were as follows: temperature, 36.7°C; pulse, 81/min; respiration, 21/min; and blood pressure, 132/67 mm Hg. The patient’s abdomen was flat, with tenderness noted in both the right upper and right lower quadrants. There was no evidence of rebound tenderness or abdominal muscle rigidity throughout the abdomen and no masses were palpated.

### 2.3. Diagnostic testing

**Figure 1.**
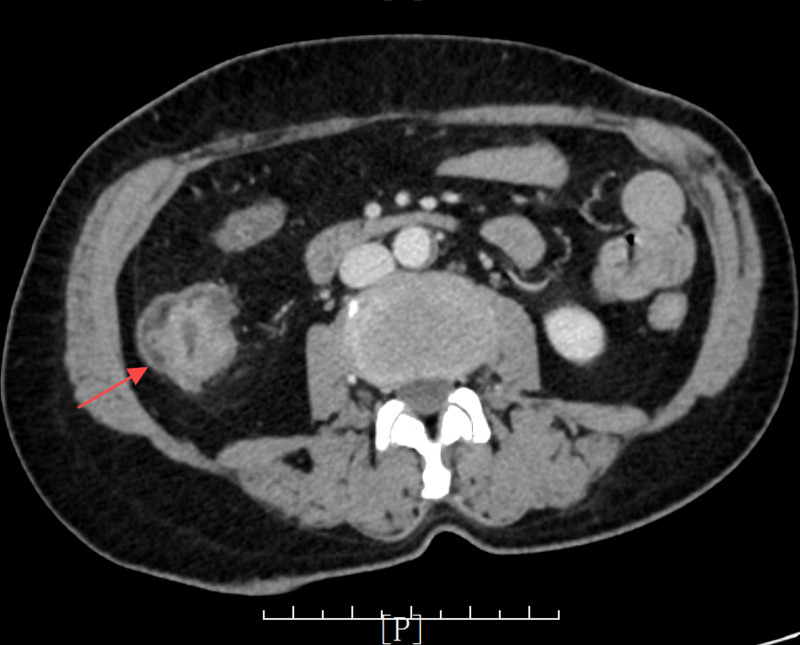
Computed tomography image (transverse section) shows ascending colon tumor (red arrow).

**Figure 2.**
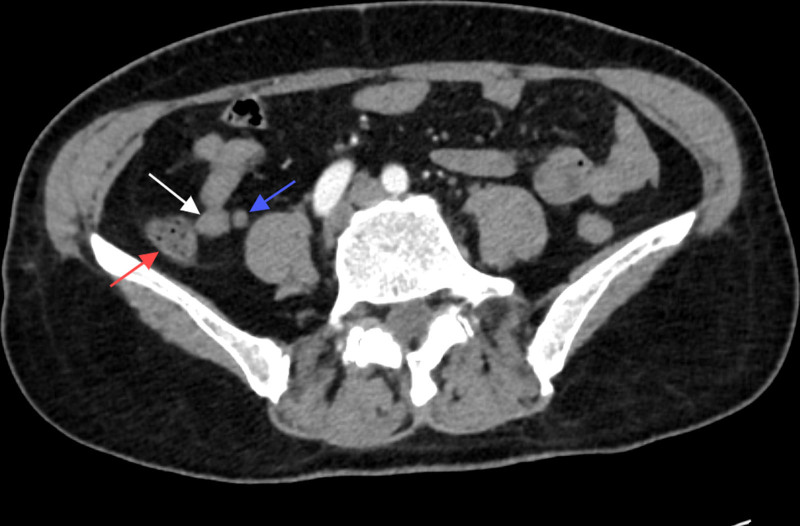
Computed tomography image (transverse section) shows a normal appendix (blue arrow), cecum (red arrow), and terminal ileum (white arrow).

**Figure 3.**
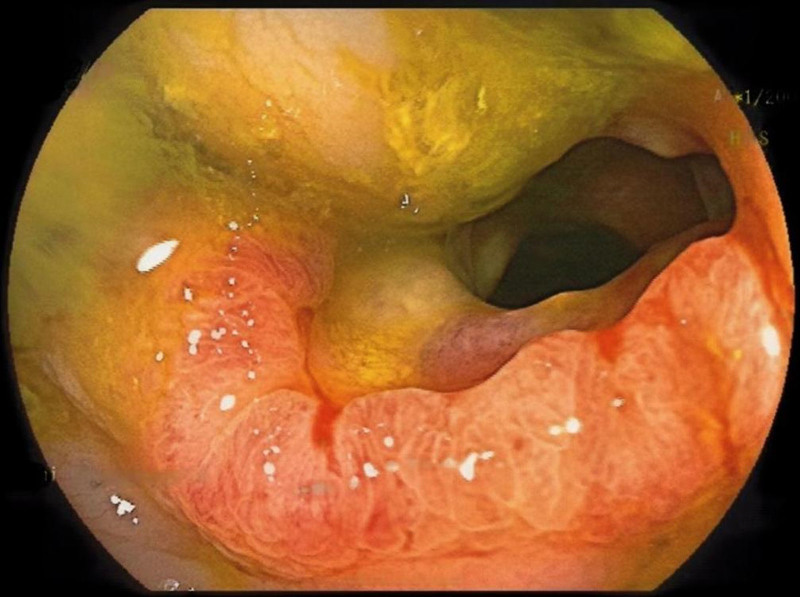
Colonoscopy image shows a tumor in the ascending colon with surface congestion and erosion.

Laboratory tests indicated a red blood cell count of 2.84 × 10^12^/L and hemoglobin levels of 55.00 g/L. Abdominal computed tomography (CT) demonstrated thickening of the ascending colon, raising suspicion of a colon tumor (Fig. 1), and no significant abnormalities were observed in the appendix (Fig. 2). Colonoscopy confirmed the presence of a mass in the ascending colon (Fig. 3), and biopsy results suggested adenocarcinoma. Combined with relevant examinations, the preoperative diagnosis was ascending colon adenocarcinoma with severe anemia.

### 2.4. Therapeutic intervention, follow-up, and outcomes

**Figure 4.**
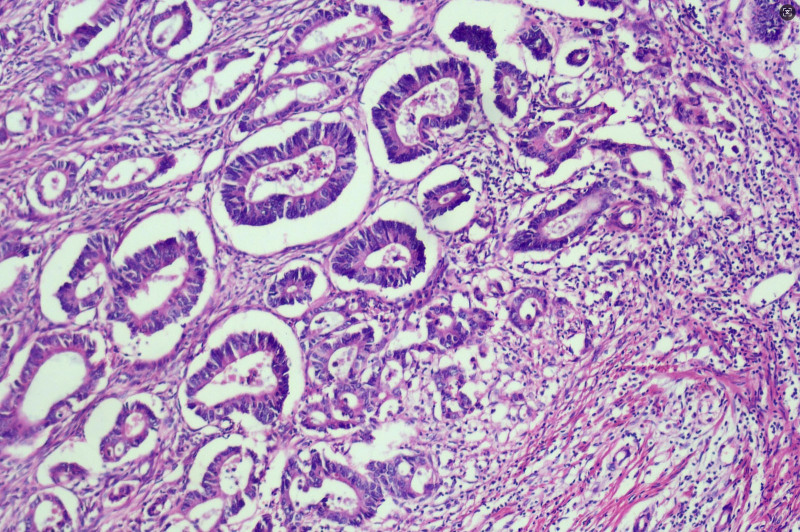
Differentiated adenocarcinoma of the ascending colon, within the muscularis propria, there are variably sized atypical glandular structures demonstrating infiltrative growth, accompanied by fibrous stromal proliferation and prominent lymphocytic infiltration (HE staining ×100 times).

**Figure 5.**
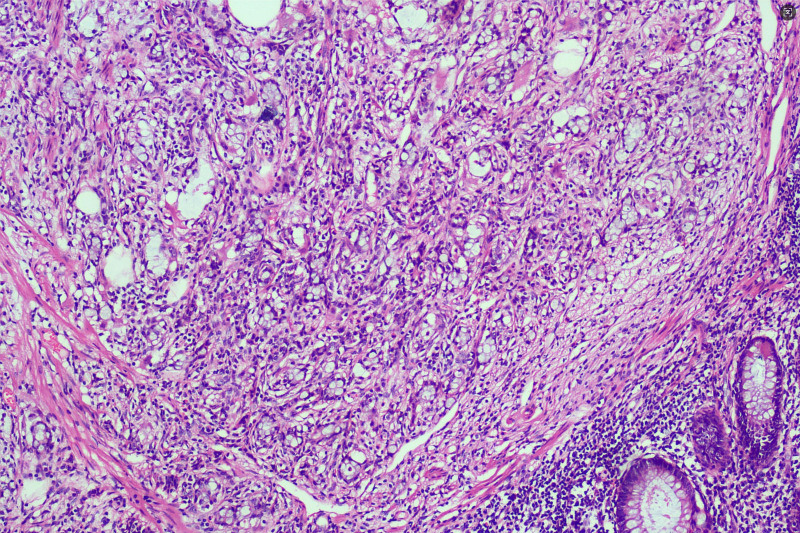
Within the muscularis propria of the appendix, there are abundant atypical cells arranged in nests, glandular-like structures, or cord-like patterns, with neoplastic cells exhibiting infiltrative growth within the muscular layer (HE staining ×100 times).

**Figure 6.**
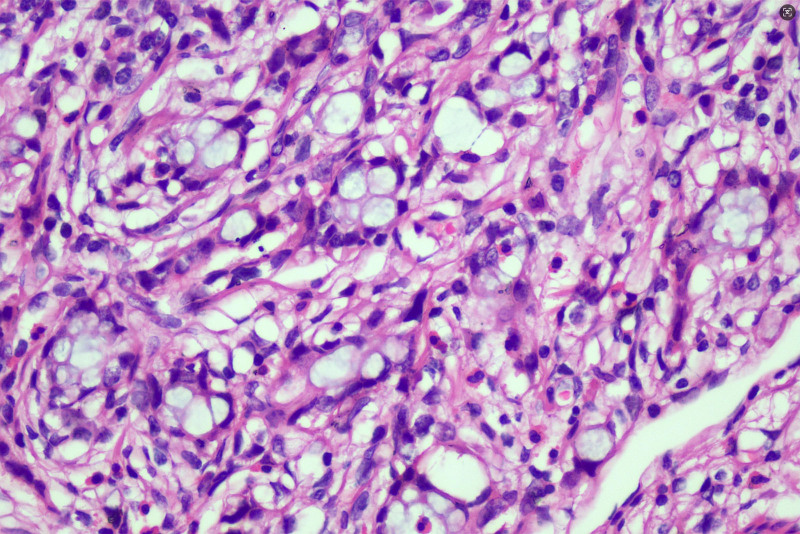
Microscopic features of appendiceal goblet cell adenocarcinoma, The neoplastic cells demonstrate marked variation in size, resembling signet-ring cells and small intestinal goblet cells, with cytoplasm distended by abundant mucin and crescent-shaped nuclei displaced to the cellular periphery (HE staining ×400 times).

On February 25, 2021, the patient underwent radical right hemicolectomy (RHC) under general anesthesia. Postoperative pathological findings were as follows: Ulcerative moderately differentiated adenocarcinoma of the ascending colon infiltrated the periintestinal adipose tissue, blood vessels, and nerves with no lymph node metastasis (0/22), within the muscularis propria, there are variably sized atypical glandular structures demonstrating infiltrative growth, accompanied by fibrous stromal proliferation and prominent lymphocytic infiltration(Fig. 4). The positive rates for cancer cell markers were MLH1(+), PMS2(+), MSH2(+), MSH6(+), P53(+), and Ki-67 (80%), Pathological TNM staging (eighth edition AJCC): T4aN1cM0 Stage IIIC. Within the muscularis propria of the appendix, there are abundant atypical cells arranged in nests, glandular-like structures, or cord-like patterns, with neoplastic cells exhibiting infiltrative growth within the muscular layer (Fig. 5), The neoplastic cells demonstrate marked variation in size, resembling signet-ring cells and small intestinal goblet cells, with cytoplasm distended by abundant mucin and crescent-shaped nuclei displaced to the cellular periphery (Fig. 6). Positivity rates for tumor markers included CEA(+), CD56(+), CgA(+), Syn(+), CD68(−), and Ki-67 (10%), Pathological TNM staging (eighth edition AJCC): T2N0M0 Stage Ⅰ. After the surgery, the patient received the standard FOLFOX6 (oxaliplatin 85 mg/m² IV over 2 hours, followed by leucovorin 400 mg/m² IV over 2 hours, then 5-FU 400 mg/m² IV bolus and 2400 to 3000 mg/m² continuous infusion over 46 hours, repeated every 14 days) chemotherapy regimen in the Department of Oncology. Upon completion of cycle 4 of chemotherapy, thoracic and abdominal CT scans combined with serum CEA and CA19-9 levels demonstrated no evidence of tumor recurrence. However, the patient discontinued further treatment due to chemotherapy-related adverse effects. Over the subsequent 46 months, multiple telephone follow-ups were conducted, during which the patient consistently denied any discomfort and reported normal bowel habits. The last follow-up on December 31, 2024 confirmed that the patient had undergone CT imaging and CEA testing at an external healthcare facility earlier in the year, with no radiologic or biochemical evidence of tumor recurrence or metastasis identified.

## 3. Discussion

AGCA is a bidirectionally differentiated malignant epithelial tumor characterized by mucinous goblet cells, varying quantities of endocrine cells, and Paneth-like cells with a granular eosinophilic cytoplasm.^[4]^ Typically, AGCA are discovered incidentally in specimens obtained following appendicitis surgery.^[5]^ Synchronous AGCA in surgical specimens of CRC is exceedingly rare. Owing to limited research in this area, the oncogenic mechanisms, diagnosis, and treatment of the coexistence of AGCA and CRC face significant challenges.^[5]^

AGCA has neuroendocrine function and was previously known as an appendiceal carcinoid. In 2019, it was redefined as adenocarcinoma in the 5th edition of the World Health Organization classification of gastrointestinal tumors.^[6]^ Carcinoid tumors are considered to be closely related to the development of secondary primary malignant tumors (SPM), especially as synchronous lesions. The “paracrine-effect theory” is considered the hypothesis that best explains the association between carcinoid tumors and SPM.^[7]^ Vincenti et al reported a case of well-differentiated adenocarcinoma of the cecum (pT1N0M0, adenomatous polyp cancer) alongside appendiceal carcinoma (pT3N0M0, stage II, high-grade), suggesting that the paracrine effect of appendiceal carcinoma leads to the development of cecal adenocarcinoma.^[3]^ However, the patient with ascending colon adenocarcinoma (pT3N0M0, stage IIA) in this report was staged later than that with AGCA (pT2N0M0, stage I, low-grade). However, this may not be explained by the paracrine effects of AGCA alone. Can AGCA be considered as the second primary cancer caused by the paracrine effect of colon cancer in this case? Moreover, long-term chronic anemia resulting from ascending colon adenocarcinoma may lead to diminished immunity, potentially serving as a basis for the occurrence of AGCA as a secondary tumor.

AGCA typically presents with no obvious clinical symptoms in its early stages, and the onset of symptoms often indicates advanced disease.^[8,9]^ The 5-year survival rate for stage I AGCA is approximately 91.1%, whereas that for stage IV is only 18.9%.^[10]^ Some researchers suggest that patients with synchronous AGCA and CRC may have a worse prognosis than those with isolated CRC.^[11]^ Unfortunately, there is currently no established method for early diagnosis of AGCA. CT and colonoscopy show limited sensitivity for early AGCA diagnosis, often requiring postoperative confirmation.^[12]^ While CT detects structural abnormalities and colonoscopy occasionally identifies atypical morphology via biopsy, both exhibit high miss rates. MRI outperforms in delineating tumor origin and local infiltration patterns, aiding surgical planning.^[13]^ Advances in molecular biomarkers, multimodal imaging technologies, and the promotion of standardized diagnostic and therapeutic protocols are progressively enhancing early detection rates, offering hope for improved prognosis.^[14,15]^

AGCAs are considered more aggressive than classical carcinoids.^[16]^ The comprehensive treatment measures for AGCA depend on the TNM staging system.^[5]^ The value of adjuvant chemotherapy for patients with early stage AGCA remains unclear, but most experts recommend adjuvant chemotherapy for lymph node-positive or intermediate-to-advanced AGCA.^[4,5]^ Systemic chemotherapy combined with 5-fluorouracil and/or cytoreductive surgery has been shown to be effective in improving the prognosis and survival of patients with advanced or recurrent disease.^[17–19]^

The current guidelines of the American Society of Colon and Rectum Surgeons and the North American Society of Neuroendocrine Oncology recommend radical RHC as the standard treatment for goblet cell carcinoma of the appendix.^[20,21]^ However, some scholars argue that it may be safe to omit RHC in certain patients with AGCA.^[22]^ We contend that for patients with AGCA combined with ascending colon adenocarcinoma, radical right hemicolectomy is a reasonable approach, as it allows for the simultaneous radical resection of tumors in both locations.

Based on this case, we propose the following recommendations and hope that the medical community will consider them: first, develop a reliable method or identify a specific marker to enhance early diagnosis rates and treatment outcomes for AGCA; second, conduct more prospective clinical studies to further elucidate the clinical management strategies for AGCA synchronous with CRC.

## 4. Conclusion

Synchronous AGCA with CRC is rare. The oncogenic mechanisms, diagnosis, and treatment of the coexistence of AGCA and CRC face significant challenges, and radical tumor resection combined with postoperative chemotherapy is an important treatment strategy. Conducting additional prospective clinical studies will enhance early diagnosis and improve clinical management.

## Acknowledgments

The authors gratefully acknowledge all staff for their kind cooperation, and thank the patients for their kind cooperation.

## Author contributions

**Conceptualization:** Yi-Hu Mao.

**Visualization:** Yi-Hu Mao, Shu-Jun Li, Qi Jia, Qian Zeng, Jian Yang, Cai-Jun Yang, Qi Pu, Ting Zhang, Xue-Ping Liu, Li Jiang.

**Writing – original draft:** Yi-Hu Mao.

**Writing – review & editing:** Yi-Hu Mao.
